# Survival Tree Analysis of Interactions Among Factors Associated With Colorectal Cancer Risk in Patients With Type 2 Diabetes: Retrospective Cohort Study

**DOI:** 10.2196/62756

**Published:** 2025-04-29

**Authors:** Sarah Tsz Yui Yau, Chi Tim Hung, Eman Yee Man Leung, Albert Lee, Eng Kiong Yeoh

**Affiliations:** 1 JC School of Public Health and Primary Care, The Chinese University of Hong Kong, 4/F, School of Public Health Building, Prince of Wales Hospital, Shatin, Hong Kong, China (Hong Kong), 852 22528790

**Keywords:** colorectal cancer, risk factor, interaction, type 2 diabetes, survival analysis, decision tree, recursive partitioning, segmentation, risk stratification, public health

## Abstract

**Background:**

Colorectal cancer (CRC) and diabetes share many common lifestyle risk factors, such as obesity. However, it remains largely unknown how different factors interact to influence the risk of CRC development among patients with diabetes.

**Objective:**

This study aimed to identify the interaction patterns among factors associated with the risk of CRC incidence among patients with diabetes.

**Methods:**

This is a retrospective cohort study conducted using electronic health records from Hong Kong. Patients who were diagnosed with type 2 diabetes and received care in general outpatient clinics between 2010 and 2019 without cancer history were included and followed up until December 2019. A conditional inference survival tree was applied to examine the interaction patterns among factors associated with the risk of CRC.

**Results:**

A total of 386,325 patients were included. During a median follow-up of 6.2 years (IQR 3.3-8.0), 4199 patients developed CRC. Patients were first partitioned into 4 age groups by increased levels of CRC risk (≤54 vs 55 to 61 vs 62 to 73 vs >73 years). Among patients aged more than 54 years, male sex was the dominant risk factor for CRC within each age stratum and the associations lessened with age. Abdominal obesity (waist-to-hip ratio >0.95) and longer duration of diabetes (median 12, IQR 7-18 vs median 4, IQR 1-11 years) were identified as key risk factor for CRC among men aged between 62 and 73 years and women aged more than 73 years, respectively.

**Conclusions:**

This study suggests the interaction patterns among age, sex, waist-to-hip ratio, and duration of diabetes on the risk of CRC incidence among patients with diabetes. Findings of the study may help identify target groups for public health intervention strategies.

## Introduction

Colorectal cancer (CRC) is the third most frequently diagnosed cancer worldwide [1]. Previous studies have shown that patients with diabetes are 1.3 times likely to develop CRC when compared with those without diabetes [2]. CRC and type 2 diabetes mellitus share many common lifestyle risk factors, such as obesity [3,4], smoking [5,6], heavy alcohol use [7,8], physical inactivity [9,10], Western dietary pattern [11-13], and processed meat [12-14].

While CRC and diabetes share many overlapping risk factors, little is known about how different risk factors interact in the presence of a pre-existing diabetes condition which may influence the risk of CRC development. For example, while older age and male sex are risk factors for many cancers including CRC, it remains less certain how the association differs by age and sex [15], and how to optimally separate patients with diabetes into subgroups by age and sex according to their risk levels. A previous meta-analysis [16] showed that men are 1.83 times likely to have CRC when compared with women. However, most CRC screening guidelines for average-risk individuals are age-based (mostly starting from 50 y [17], or 45 y at the earliest [18]). It remains largely unknown how the risk levels differentiate by age and sex within the population with diabetes. Several studies have incorporated age-sex [19,20] and diabetes [19] into their proposed screening strategies. Furthermore, while adiposity is a common risk factor for diabetes and CRC [3,21], most studies focused on general obesity [3,22]. It is less clear whether general obesity may adequately represent abdominal obesity in predicting CRC risk [22-24], and whether adiposity becomes a predominant risk factor in certain subgroups of patients. A previous meta-analysis [22] found that when comparing highest with lowest categories of waist-to-hip ratio, men and women in the highest categories of waist-to-hip ratio are 1.47 and 1.3 times likely to have CRC respectively when compared with their same-sex counterparts in the lowest category. Furthermore, it is less understood whether duration of diabetes [15] or antidiabetic drug treatment [21] may influence the risk of CRC development, given the interactions between gut microbiota with obesity [25,26], insulin resistance [25,27], diabetes [25], antidiabetic drugs [25], diet [25,26], immunity [25,26], and CRC [26]. Previous studies [15,28-30] demonstrated mixed findings on the associations between duration of diabetes and CRC risk.

Previous research suggests type 2 diabetes as a heterogeneous disease [31-35], and various approaches such as clustering techniques [31-34] have been applied to explore the subtypes. For example, Ahlqvist et al [31] has used 6 variables (including age, BMI, and HbA_1c_) to stratify patients with adult-onset diabetes into 5 distinct clusters, namely “severe autoimmune diabetes”, “severe insulin-deficient diabetes”, “severe insulin-resistant diabetes”, “mild obesity-related diabetes”, and “mild age-related diabetes”, where the latter 4 clusters may represent different subtypes within type 2 diabetes. Identifying subtypes of type 2 diabetes may help individualize most appropriate treatment to help improve clinical outcomes [31,33]. Furthermore, the interplay between genetic and environmental factors [36] may collectively contribute to the risk of diabetes and CRC. A previous meta-analysis on gene-environment interaction studies found insufficient evidence for the interaction effects between genetic and environmental factors [37]. However, a recent large-scale gene-environment interaction study [38] suggested that common genetic variants related to insulin signaling and immune function may potentially modify the association between diabetes and CRC risk. In addition, some medical conditions may share common pathophysiology with diabetes. For example, obesity, immune-mediated inflammatory bowel diseases, and metabolic disorders [39] are characterized by systematic low-grade inflammation, which may in turn promote CRC development.

Given the potential complex interplay among interrelated factors for diabetes and CRC, in this study, tree-structured survival analysis is applied to examine the potential interaction patterns among a set of covariates on CRC risk. Conditional inference survival tree [40] is a tree-structured (or recursive partitioning) algorithm embedded with statistical theory of conditional inference. Tree-structured algorithms are able to account for multicollinearity among the set of covariates, and explore the potential interactions among more than 2 covariates without an exhaustive search of all possible combinations. Compared with other tree-structured algorithms, conditional inference survival tree has the advantages of (1) incorporating a theoretical framework, (2) avoiding overfitting, (3) minimizing selection bias toward covariates with many possible values, and (4) not requiring explicit pruning [40].

This study aims to examine whether there are interaction patterns among factors associated with the risk of CRC incidence among patients with pre-existing type 2 diabetes using survival tree analysis.

## Methods

### Study Design and Study Population

This is a retrospective cohort study performed using territory-wide electronic health records of Hong Kong. The Hospital Authority (HA) is statutory body managing 43 public hospitals, 49 specialist outpatient clinics, and 74 general outpatient clinics over the territory. The HA maintains a centralized data repository that stores information on patients’ demographics, disease diagnoses, prescription records, laboratory measurements, as well as inpatient and outpatient visits. Since the HA provides approximately 90% specialist and inpatient care [41], most of the cancer cases were captured in the records system. Disease diagnoses were coded with the *International Classification of Diseases, Ninth Revision* (*ICD-9*) or *International Classification of Diseases, Tenth Revision* (*ICD-10*) or the *International Classification of Primary Care, Second Edition* (*ICPC-2*). Data were accessed through HA Data Collaboration Lab.

### Patients

Patients who (1) were diagnosed with diabetes and (2) received a first diabetes complication screening assessment at any of the general outpatient clinics between 2010 and 2019 were initially included. Patients who (1) were diagnosed with non–type 2 diabetes, (2) had a missing date of diabetes diagnosis, (3) were diagnosed with diabetes below the age of 18 years, or (4) had a history of malignancy were excluded. Index date was defined as date of the first assessment. Patients were followed up until a cancer diagnosis, death, or December 31, 2019, whichever occurred earlier. Since the diagnosis of one cancer may influence the diagnosis of another cancer [42], those who received a cancer diagnosis at sites other than colon or rectum during follow-up were excluded. In addition, to minimize reverse causality, those who had less than 6 months of follow-up [42] were also excluded.

The diagnosis of type 2 diabetes was based on *ICPC-2* code (T90), and defined as a clinical diagnosis by clinicians, with 2 abnormal test results of plasma glucose and presentation of clinical symptoms. Type 2 diabetes is pharmaceutically treated by glucose lowering agents (most commonly used drugs are metformin, sulphonylurea, and insulin, since these drugs are less expensive options) [41]. HbA_1c_ is the primary measure used to monitor blood glucose control.

### Outcome

The outcome of interest was diagnosis of CRC (*ICD-9*: 153‐154; *ICD-10*: C18-21) during follow-up. Patients who presented with CRC symptoms (such as rectal bleeding or blood in stool) were recommended to take a fecal occult blood test. If the results were positive, patients would be referred for a diagnostic colonoscopy procedure. For CRC diagnosis, gastroenterologists would perform a colonoscopy procedure to remove polyps and take biopsy samples for further examination by pathologists to provide a diagnosis. CRC are generally classified into 4 consensus molecular subtypes (CMSs), namely CMS1 (microsatellite instability immune*)*, CMS2 (canonical*)*, CMS3 (metabolic*)*, and CMS4 (mesenchymal*)*, with distinct characteristics and developmental pathways [43,44]. Possible treatment options include surgery, radiotherapy, chemotherapy, and targeted therapy.

### Covariates

Information on input data was ascertained during the first assessment. Candidate split variables included demographics (age and sex), duration of diabetes, medical history (ischemic heart disease, cerebrovascular disease, heart failure, hypertension, chronic kidney disease, liver cirrhosis, chronic obstructive pulmonary disease, pneumonia, and family history of diabetes), medication use (antidiabetic drugs, aspirin, nonsteroidal anti-inflammatory drugs, anticoagulants, antiplatelets, antihypertensive drugs, and statins), lifestyle behaviors (alcohol use and smoking), anthropometric measurements (waist-to-hip ratio and BMI), and laboratory measurements (HbA_1c_, fasting glucose, low-density lipoprotein cholesterol, high-density lipoprotein cholesterol, triglycerides, and serum creatinine). Duration of diabetes was the time difference between diabetes diagnosis and the first assessment. Medication use was defined as whether patients had been prescribed a drug at the time of the assessment. Antidiabetic drugs included were metformin, sulfonylurea, insulin, and dipeptidyl peptidase-4 inhibitors. Laboratory measurements were taken from most recent results to the assessment date.

### Data Analysis

Conditional inference survival tree [40] was applied to examine the interaction patterns among factors associated with the risk of CRC incidence. At each split, a global null hypothesis of independence between any of the candidate split variables and the risk of CRC was first tested at a prespecified α level. If rejected, a set of partial null hypotheses of independence between each covariate and the risk of CRC were then tested, and the covariate with strongest association or smallest Bonferroni-corrected *P* value would be selected as split variable. The partitioning procedures were recursively conducted until the global null hypothesis cannot be rejected. For continuous variables, the cutoff point was the optimal value to maximize the between-node differences in survival probability. The statistical significance threshold α level and maximum depth of the survival tree were set at .01 and 4, respectively. Each path from the root node to a terminal node represents an interaction pattern [45]. The effects of a split variable are conditional on split variables chosen at its ancestor nodes. Patients were partitioned into mutually exclusive subgroups at terminal nodes with most homogenous within-group survival outcomes. The CRC-free survival of subgroups of patients with diabetes was examined using Kaplan–Meier method. Model performance was evaluated using area under the curve as metric. In post hoc analyses, the associations between identified important factors and the risk of CRC were estimated using Cox regression and reported in adjusted hazard ratio (aHR) with 95% CI. Data analyses were performed using R software (version 4.2.3; R Foundation for Statistical Computing).

### Ethical Considerations

Ethics approval for secondary data analysis was provided by the Chinese University of Hong Kong – Survey and Behavioural Research Ethics Committee (SBRE-22-0386). Patient consent was waived since individuals were not identifiable in this study.

## Results

### Overview

**Figure 1.**
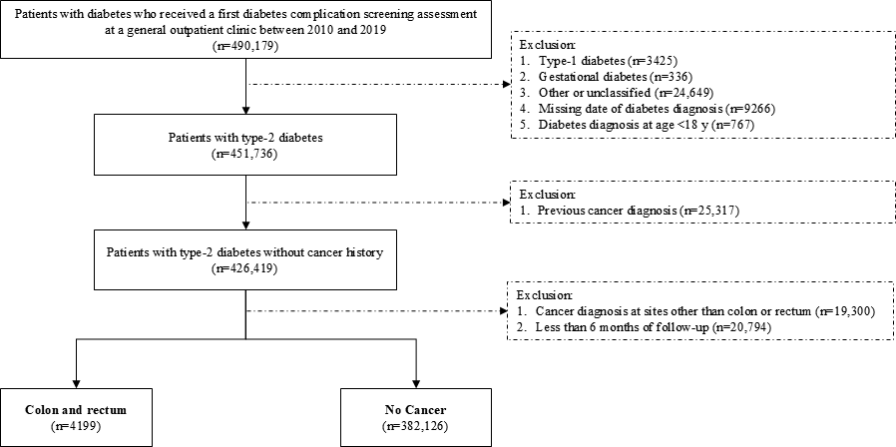
Flowchart of patient selection.

**Figure 2.**
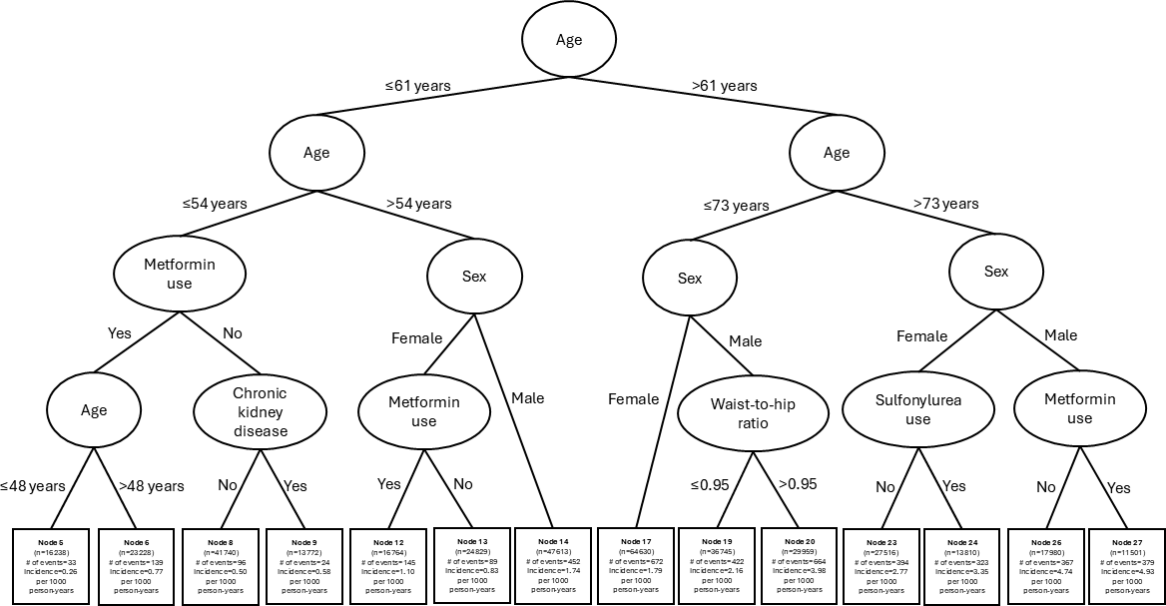
Survival tree diagram illustrating the interaction patterns among key factors on colorectal cancer incidence among patients with diabetes.

Figure 1 shows the flowchart of patient selection. Of the 386,325 patients included, 4199 patients developed CRC during a median follow-up of 6.2 (IQR 3.3-8.0) years. The incidence rates among female and male patients aged more than 54 years were 1.90 and 2.94 per 1000 person-years, respectively. On the other hand, the incidence rate among patients aged ≤54 years across both sexes was 0.54 per 1000 person-years. The survival tree first partitioned patients into 4 age groups (≤54 vs 55 to 61 vs 62 to 73 vs >73 years) by increasing levels of CRC risk. Among patients aged more than 54 years, male sex was identified as most dominant risk factor for CRC within each age stratum. Waist-to-hip ratio and sulfonylurea use (characterized by long duration of diabetes) emerged as important factor in differentiating the risk of CRC among men aged between 62 and 73 years and women aged more than 73 years, respectively (Figure 2 and Tables1  2).

**Table 1.** Characteristics of different subgroups of patients with diabetes.

Characteristics	Node (5,6,8,9)	Node (12,13)	Node14	Node17	Node19	Node20	Node23	Node24	Node (26,27)
	(n=94,978)	(n=41,593)	(n=47,613)	(n=64,630)	(n=36,745)	(n=29,959)	(n=27,516)	(n=13,810)	(n=29,481)
Colorectal cancer cases during follow (up, n (%)	292 (0.31)	234 (0.56)	452 (0.95)	672 (1.04)	422 (1.15)	664 (2.22)	394 (1.43)	323 (2.34)	746 (2.53)
Demographics									
Male, n (%)	53,228 (56.04)	0 (0)	47,613 (100)	0 (0)	36,745 (100)	29,959 (100)	0 (0)	0 (0)	29,481 (100)
Age at assessment (y), mean (SD)	47.68 (6.04)	58.1 (2.00)	58.09 (2.00)	66.95 (3.40)	66.67 (3.36)	66.91 (3.38)	79.85 (4.58)	79.52 (4.35)	79.03 (4.21)
Duration of diabetes (y), median (IQR)	2 (0‐5)	2 (1-7)	3 (1-8)	4 (1-10)	4 (1-10)	3 (1-9)	4 (1-11)	12 (7-18)	6 (1-12)
Medical history									
Ischemic heart disease, n (%)	3096 (3.26)	1026 (2.47)	3979 (8.36)	3625 (5.61)	4135 (11.25)	3351 (11.19)	2908 (10.57)	1500 (10.86)	4519 (15.33)
Cerebrovascular disease, n (%)	2353 (2.48)	1378 (3.31)	2501 (5.25)	3579 (5.54)	3062 (8.33)	2496 (8.33)	3086 (11.22)	1396 (10.11)	4108 (13.93)
Heart failure, n (%)	827 (0.87)	326 (0.78)	720 (1.51)	1005 (1.56)	753 (2.05)	708 (2.36)	1373 (4.99)	665 (4.82)	1470 (4.99)
Hypertension, n (%)	69,712 (73.40)	33,998 (81.74)	39,564 (83.09)	58,252 (90.13)	31,876 (86.75)	27,702 (92.47)	26,271 (95.48)	13,405 (97.07)	27,904 (94.65)
Chronic kidney disease, n (%)	13,772 (14.50)	4563 (10.97)	7806 (16.39)	8112 (12.55)	6529 (17.77)	5213 (17.40)	6748 (24.52)	0 (0)	5971 (20.25)
Liver cirrhosis, n (%)	2376 (2.50)	732 (1.76)	1123 (2.36)	1159 (1.79)	653 (1.78)	626 (2.09)	373 (1.36)	193 (1.40)	430 (1.46)
Chronic obstructive pulmonary disease, n (%)	75 (0.08)	33 (0.08)	206 (0.43)	139 (0.22)	453 (1.23)	475 (1.59)	251 (0.91)	97 (0.70)	1087 (3.69)
Pneumonia, n (%)	1695 (1.78)	582 (1.40)	1158 (2.43)	1402 (2.17)	1260 (3.43)	1084 (3.62)	1656 (6.02)	761 (5.51)	2511 (8.52)
Family history of diabetes, n (%)	59,255 (62.39)	23,293 (56.00)	25,487 (53.53)	28,911 (44.73)	15,127 (41.17)	11,406 (38.07)	7260 (26.38)	4412 (31.95)	7062 (23.95)
Lifestyle behaviors									
Current drinker or ex-drinker, n (%)	32,141 (33.84)	5011 (12.05)	23,558 (49.48)	6130 (9.48)	16,429 (44.71)	14,456 (48.25)	2082 (7.57)	1001 (7.25)	11,157 (37.84)
Current smoker or ex-smoker, n (%)	31,727 (33.40)	1951 (4.69)	24,514 (51.49)	2596 (4.02)	19,260 (52.42)	17,033 (56.85)	1781 (6.47)	934 (6.76)	16,182 (54.89)
Anthropometric measurements									
Waist-to-hip ratio, mean (SD)	0.93 (0.06)	0.92 (0.06)	0.95 (0.05)	0.93 (0.06)	0.92 (0.03)	1 (0.04)	0.94 (0.07)	0.94 (0.07)	0.96 (0.06)
Body mass index (kg/m^2^), mean (SD)	27.26 (4.82)	26.16 (4.33)	26.16 (3.82)	25.81 (4.10)	24.52 (3.23)	26.87 (3.42)	25.31 (3.87)	24.95 (3.73)	24.95 (3.29)
Laboratory measurements									
HbA_1c_ (%), mean (SD)	7.62 (1.72)	7.38 (1.40)	7.47 (1.56)	7.24 (1.24)	7.27 (1.42)	7.34 (1.37)	6.96 (1.09)	7.32 (1.15)	7.13 (1.24)
Fasting glucose (mmol/L), mean (SD)	8 (2.60)	7.65 (2.27)	7.78 (2.37)	7.45 (2.01)	7.51 (2.16)	7.55 (2.13)	7.15 (1.79)	7.39 (2.05)	7.23 (1.90)
Low-density lipoprotein cholesterol (mmol/L), mean (SD)	2.77 (0.83)	2.84 (0.86)	2.66 (0.81)	2.71 (0.84)	2.55 (0.79)	2.57 (0.79)	2.59 (0.83)	2.64 (0.79)	2.47 (0.76)
High-density lipoprotein cholesterol (mmol/L), mean (SD)	1.21 (0.31)	1.36 (0.34)	1.19 (0.30)	1.36 (0.34)	1.25 (0.33)	1.15 (0.28)	1.39 (0.36)	1.32 (0.35)	1.23 (0.33)
Triglycerides (mmol/L), mean (SD)	1.81 (1.56)	1.62 (1.06)	1.65 (1.26)	1.59 (0.94)	1.39 (0.96)	1.62 (1.10)	1.54 (0.83)	1.53 (0.87)	1.34 (0.81)
Serum creatinine (µmol/L), mean (SD)	75.2 (42.11)	65.7 (29.00)	88.52 (45.86)	71.79 (32.63)	93.11 (44.86)	95.17 (43.13)	82.34 (37.03)	86.73 (34.08)	106.12 (44.41)

**Table 2.** Medication use of different subgroups of patients with diabetes.

	Node (5,6,8,9)	Node (12,13)	Node14	Node17	Node19	Node20	Node23	Node24	Node (26,27)
	(n=94,978)	(n=41,593)	(n=47,613)	(n=64,630)	(n=36,745)	(n=29,959)	(n=27,516)	(n=13,810)	(n=29,481)
Antidiabetic drugs, n (%)									
Metformin	39,466 (41.55)	16,764 (40.30)	18,438 (38.72)	26,945 (41.69)	13,084 (35.61)	12,524 (41.80)	8271 (30.06)	10,160 (73.57)	11,501 (39.01)
Sulfonylurea	23,445 (24.68)	10,078 (24.23)	12,184 (25.59)	17,740 (27.45)	9315 (25.35)	8905 (29.72)	0 (0)	13,810 (100)	9608 (32.59)
Insulin	6460 (6.80)	2154 (5.18)	2983 (6.27)	3703 (5.73)	2362 (6.43)	1991 (6.65)	1801 (6.55)	541 (3.92)	2053 (6.96)
Dipeptidyl peptidase-4 inhibitors	3497 (3.68)	1292 (3.11)	2006 (4.21)	2383 (3.69)	1671 (4.55)	1134 (3.79)	1059 (3.85)	481 (3.48)	1302 (4.42)
Sodium-glucose cotransporter-2 inhibitors	371 (0.39)	77 (0.19)	215 (0.45)	115 (0.18)	154 (0.42)	46 (0.15)	30 (0.11)	0 (0)	35 (0.12)
Glucagon-like peptide-1 receptor agonists	123 (0.13)	18 (0.04)	26 (0.05)	11 (0.02)	4 (0.01)	11 (0.04)	2 (0.01)	0 (0)	2 (0.01)
Glucosidase inhibitors	308 (0.32)	155 (0.37)	181 (0.38)	301 (0.47)	128 (0.35)	145 (0.48)	45 (0.16)	146 (1.06)	165 (0.56)
Meglitinide	35 (0.04)	20 (0.05)	15 (0.03)	27 (0.04)	10 (0.03)	9 (0.03)	9 (0.03)	7 (0.05)	15 (0.05)
Glitazone	463 (0.49)	144 (0.35)	153 (0.32)	245 (0.38)	91 (0.25)	131 (0.44)	19 (0.07)	90 (0.65)	91 (0.31)
Any of the above	51,272 (53.98)	20,504 (49.30)	24,369 (51.18)	33,517 (51.86)	18,012 (49.02)	15,998 (53.40)	10,366 (37.67)	13,810 (100)	16,343 (55.44)
Aspirin, n (%)	374 (0.39)	4591 (11.04)	9924 (20.84)	12,660 (19.59)	10,418 (28.35)	8628 (28.80)	9160 (33.29)	4323 (31.30)	11,498 (39.00)
Nonsteroidal anti-inflammatory drugs, n (%)	49,797 (52.43)	26,009 (62.53)	24,009 (50.43)	38,648 (59.80)	17,489 (47.60)	15,470 (51.64)	15,693 (57.03)	7137 (51.68)	13,781 (46.75)
Anticoagulants, n (%)	2816 (2.96)	910 (2.19)	2636 (5.54)	2513 (3.89)	2704 (7.36)	2021 (6.75)	2024 (7.36)	759 (5.50)	2651 (8.99)
Antiplatelets, n (%)	3381 (3.56)	1429 (3.44)	4104 (8.62)	3854 (5.96)	470 (1.28)	2826 (9.43)	3687 (13.40)	0 (0)	3728 (12.65)
Antihypertensive drugs, n (%)	48,987 (51.58)	26,534 (63.79)	30,941 (64.98)	48,850 (75.58)	25,567 (69.58)	24,265 (80.99)	23,146 (84.12)	12,537 (90.78)	24,676 (83.70)
Statins, n (%)	37,282 (39.25)	20,845 (50.12)	24,015 (50.44)	36,367 (56.27)	19,802 (53.89)	16,520 (55.14)	15,871 (57.68)	5939 (43.01)	15,378 (52.16)

### Age and Sex

**Figure 3.**
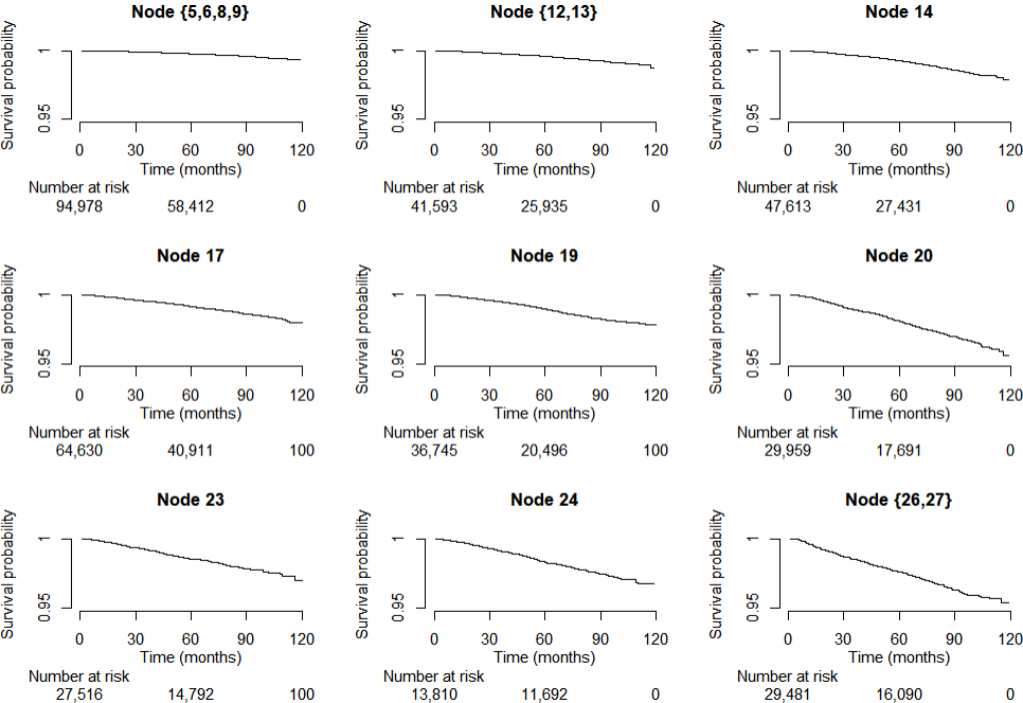
Kaplan–Meier curves for colorectal cancer-free survival across distinct subgroups of patients with diabetes.

The optimal cutoffs identified to differentiate the increasing levels of CRC risk with age were 54, 61, and 73 years (Figure 2 and Figure 3) . Among patients aged more than 54 years, male sex was the most important risk factor for CRC within each age group (Figure 2). Men had an elevated risk of developing CRC when compared with women within each age stratum (for 55 to 61 years: aHR 1.81, 95% CI 1.54‐2.12; for 62 to 73 years: aHR 1.70, 95% CI 1.54‐1.87; and for >73 years: aHR 1.64, 95% CI 1.48‐1.82), after controlling for age and duration of diabetes. On the other hand, among the youngest age group (≤54 years), no split variable was identified to have individually significant effects on the risk of CRC after adjusting for age, sex, and duration of diabetes (Table 3).

**Table 3.** Comparisons in adjusted hazard ratios of split variables between comparison nodes at the third level (comparison 1 was adjusted for age, sex, and duration of diabetes; and comparisons 2 to 4 were controlled for age and duration of diabetes).

Node	Characteristics	Comparison 1,metformin use in the youngestaHR^a^ (95% CI)	Comparison 2,sex in middle-youngaHR (95% CI)	Comparison 3,sex in middle-oldaHR (95% CI)	Comparison 4,sex in the oldestaHR (95% CI)
Node (5,6)	Metformin users aged ≤54 years	1 (reference)	—^b^	—	—
Node (8,9)	Metformin nonusers aged ≤54 years	1.15 (0.90‐1.47)	—	—	—
Node (12,13)	Female aged between 55 and 61 years	—	1 (reference)	—	—
Node 14	Male aged between 55 and 61 years	—	1.81 (1.54‐2.12)	—	—
Node 17	Female aged between 62 and 73 years	—	—	1 (reference)	—
Node (19,20)	Male aged between 62 and 73 years	—	—	1.70 (1.54‐1.87)	—
Node (23,24)	Female aged more than 73 years	—	—	—	1 (reference)
Node (26,27)	Male aged more than 73 years	—	—	—	1.64 (1.48‐1.82)

a aHR: adjusted hazard ratio.

b Not available.

### Age, Sex, and Waist-to-Hip Ratio

Among male patients aged between 62 and 73 years, elevated waist-to-hip ratio (>0.95) appeared as dominant risk factor for CRC (Figure 2). Those with a ratio of 0.95 above had an increased risk of developing CRC (aHR 1.82, 95% CI 1.61‐2.05) when compared with those with a ratio ≤0.95, when controlling for age and duration of diabetes (Table 4). The corresponding mean waist-to-hip ratios of these 2 subgroups were 1.00 (SD 0.04) and 0.92 (SD 0.03).

**Table 4.** Comparisons in adjusted hazard ratios of split variables between sibling nodes at the bottom level (comparisons 1 to 2 were made with adjustment of age, sex, and duration of diabetes; comparisons 3, 4, and 6 were adjusted for age and duration of diabetes; and comparison 5 was adjusted for age, duration of diabetes, HbA_1c_ and fasting glucose).

Node	Characteristics	Comparison 1, age in the youngest, aHR^a^ (95% CI)	Comparison 2, chronic kidney disease in the youngest, aHR (95% CI)	Comparison 3, metformin use in the middle-young femaleaHR (95% CI)	Comparison 4, waist-to-hip ratio in the middle-old maleaHR (95% CI)	Comparison 5, sulfonylurea use in the oldest femaleaHR (95% CI)	Comparison 6, metformin use in the oldest maleaHR (95% CI)
Node 5	Metformin users aged ≤48 years	1 (reference)	—^b^	—	—	—	—
Node 6	Metformin users aged between 49 and 54 years	1.45 (0.77‐2.71)	—	—	—	—	—
Node 8	Metformin nonusers aged ≤54 years without chronic kidney disease	—	1 (reference)	—	—	—	—
Node 9	Metformin nonusers aged ≤54 years with chronic kidney disease	—	1.43 (0.89‐2.29)	—	—	—	—
Node 12	Female metformin users aged between 55 and 61 years	—	—	1.06 (0.80‐1.40)	—	—	—
Node 13	Female metformin nonusers aged between 55 and 61 years	—	—	1 (reference)	—	—	—
Node 19	Male aged between 62 and 73 years with waist-to-hip ratio ≤0.95	—	—	—	1 (reference)	—	—
Node 20	Male aged between 62 and 73 years with waist-to-hip ratio >0.95	—	—	—	1.82 (1.61‐2.05)	—	—
Node 23	Female sulfonylurea nonusers aged >73 years	—	—	—	—	1 (reference)	—
Node 24	Female sulfonylurea users aged>73 years	—	—	—	—	1.19 (1.02‐1.39)	—
Node 26	Male metformin nonusers aged >73 years	—	—	—	—	—	1 (reference)
Node 27	Male metformin users aged >73 years	—	—	—	—	—	1.03 (0.89‐1.19)

a aHR: adjusted hazard ratio.

b Not available.

### Age, Sex, and Duration of Diabetes

Among female patients aged more than 73 years, sulfonylurea use emerged as key factor in differentiating the risk of CRC (Figure 2). Those who had been prescribed sulfonylurea were characterized by long duration of diabetes when compared with those who were not prescribed with sulfonylurea (median 12, IQR 7-18 vs median 4, IQR 1-11 years). Among the oldest female group, sulfonylurea users had a higher risk of developing CRC (aHR 1.19, 95% CI 1.02‐1.39) when compared with sulfonylurea nonusers, controlling for age, duration of diabetes, HbA_1c_, and fasting glucose (Table 4).

### Model Performance

The areas under the curve of the tree model at 2, 5, and 7 years were 0.713, 0.696, and 0.685, respectively.

## Discussion

### Principal Findings

This study adopted a supervised learning algorithm approach, namely conditional inference survival tree embedded with statistical theory, to identifying key factors and their optimal cutoffs to differentiate the risk of CRC and separate patients with type 2 diabetes into mutually exclusive subgroups of most homogenous CRC risk. Age, sex, waist-to-hip ratio, and sulfonylurea use exhibited interaction patterns on the risk of CRC incidence among the study diabetes cohort. Despite older age, male sex, and obesity being established individual risk factors for CRC, the optimal cutoffs for age among the study cohort were identified to partition patients according to their CRC risk levels using a data-driven approach. Furthermore, while male sex emerged as most important risk factor among patients aged more than 54 years, abdominal obesity appeared as dominant risk factor among men aged 62 to 73 years. In addition, sulfonylurea use (as characterized by long duration of diabetes) was identified as key factor in differentiating CRC risk among oldest women aged more than 73 years.

This study found that male sex emerged as dominant risk factor for CRC among patients aged more than 54 years and the associations weakened with age, implying the potentially differential associations between male sex and CRC risk with age. A previous meta-analysis [16] found that the associations between male sex and CRC risk grew markedly from age 50 years but dropped gradually after reaching 60 years. A similar trend was observed in this study, where male sex was found to be an important risk factor from 55 years onwards, and the associations declined with age. In a more recent study performed among young adults who received colonoscopies in South Korea [46], male sex was only found to be a significant risk factor for overall colorectal neoplasia among patients aged 30 to 39 years, but not among those aged between 20 and 29 years. However, male sex was not associated with advanced colorectal neoplasia within each age group [46]. In another study conducted among patients who underwent colonoscopies in Korea [47], male sex was found to be a risk factor for colorectal adenoma among the overall cohort, but not younger patients aged less than 50 years. Furthermore, epigenomic evidence supports that the second period of abrupt changes in immunity occurs earlier in men than in women (early 60s vs late 60s), and the changes are more pronounced in men [48]. The discrepancy (5 to 6 years) in second period of accelerated aging coincides with life expectancy of men and women [48]. This change in immunity could partially explain the dominance of male sex on CRC risk starting from middle adulthood, and the effects decline over time as both sexes undergo rapid changes.

Furthermore, the survival tree model selected abdominal obesity (waist-to-hip ratio >0.95) over general obesity (BMI) as a dominant risk factor among middle-old men aged between 62 and 73 years. In a previous study conducted among patients who received colonoscopies in Korea [49], abdominal obesity was only found to be significantly associated with the risk of colorectal neoplasia in men but not women. Several reviews also summarized that obesity has a stronger effect on the risk of CRC among men than women [50-52], and weight gain later in life is a key risk factor for CRC among men [50,51]. Physiologically, men are predisposed to accumulating visceral fat than women [53], and this could account for the stronger link between obesity and CRC in men. The dominance of abdominal obesity as key risk factor among middle-old men could be due to decline in testosterone with age. Previous research has shown that lower testosterone in men is linked to abdominal obesity and metabolic abnormalities [51,53]. Furthermore, individuals tend to accumulate visceral fat and lose skeletal muscle with age, rendering general obesity being not sufficiently representative of abdominal obesity among older individuals [54].

In addition, oldest women aged more than 73 years who took sulfonylurea and had a longer duration of diabetes (median 12, IQR 7-18 years) appeared to have a greater risk of CRC risk than oldest women who did not take sulfonylurea and had a shorter duration of diabetes (median 4, IQR 1-11 years) in this study. It is unclear whether the elevated risk is due to longer duration of diabetes or sulfonylurea use. In the existing literature, the association between sulfonylurea use and cancer risk remains controversial. Previous studies generally reported a positive association [21] while some did not find any association [55,56]. In a study performed in Korea [57], sulfonylurea use was only found to be associated with an increased risk of CRC among patients aged 65 years or older, but not among those below the age of 65 years. In this study, sulfonylurea use only appeared to be a key factor associated with an increased risk of CRC among women aged more than 73 years. Given sulfonylurea users being characterized by long duration of diabetes, it is possible that changing gut microbiota over the course of diabetes progression [25,27] may eventually influence carcinogenesis of the colon and rectum [26].

This study identified the optimal cutoffs for age to partition patients with type 2 diabetes in the study cohort by their risk levels of CRC using a data-driven approach. While male sex is known to be a risk factor for many cancers, including CRC, the association between male sex and CRC appears to be more apparent among older patients with diabetes (aged more than 54 years). Nevertheless, the links become less strong with age. Furthermore, abdominal obesity becomes a dominant risk factor over general obesity among male patients with diabetes aged between 62 and 73 years. In addition, women aged more than 73 years with a long duration of diabetes could be potentially at greater risk of CRC than their counterparts with a shorter duration of diabetes. Findings of the study may offer valuable insights into identifying profiles of potential target groups for future public health interventions.

Strengths of this study include incorporation of a diabetes cohort with a relatively large sample size, regular follow-up, and routine surveillance, availability of information from electronic health records, and adoption of a supervised learning algorithm such that selection of key factors is directly driven by outcome. However, several limitations may exist. First, information on some potential confounders such as dietary exposure, family history of CRC, insulin, or C-peptide was not available. Second, dosage and duration of medication use was not examined. Third, different types of sulfonylureas were not differentially evaluated in this study. Finally, the dominance of key factors and optimal cutoffs may vary across different populations. Future research is warranted to verify the findings. Examples include validation of optimal age-sex segmentation for CRC risk in a prospective cohort, incorporation of a comparison group without diabetes, exploration of the role of adiposity (general and abdominal) in CRC risk over the life course across sexes in a prospective cohort with a long-term follow-up, and new-user design in exploring drug effects (such as commonly used antidiabetic drugs and newer drugs including semaglutide) on CRC risk. Reproducible results of identified subtypes of type 2 diabetes associated with differential CRC risk may help guide public health intervention strategies and optimize clinical outcomes [33].

### Conclusions

This study suggested the potential interaction patterns among age, sex, abdominal obesity, and duration of diabetes on the risk of CRC among patients with diabetes. While older age, male sex, and obesity are well-established risk factors for CRC, the combinations of risk factors may differentially contribute to different levels of CRC risk among patients with diabetes. Findings of the study may potentially help identify target groups for public health intervention strategies.

## Abbreviations

aHR: adjusted hazard ratio
CMS: consensus molecular subtype
CRC: colorectal cancer
HA: Hospital Authority
*ICD-10*: *Interactional Classification of Diseases, Tenth Revision*
*ICD-9*: *Interactional Classification of Diseases, Ninth Revision*
*ICPC-2*: *International Classification of Primary Care, Second Edition*
